# Identification of a high incidence region for retroviral vector integration near exon 1 of the *LMO2 *locus

**DOI:** 10.1186/1742-4690-6-79

**Published:** 2009-09-02

**Authors:** Koichiro Yamada, Tomonori Tsukahara, Kazuhisa Yoshino, Katsuhiko Kojima, Hideyuki Agawa, Yuki Yamashita, Yuji Amano, Mariko Hatta, Yasunori Matsuzaki, Naoki Kurotori, Keiko Wakui, Yoshimitsu Fukushima, Ryosuke Osada, Tanri Shiozawa, Kazuo Sakashita, Kenichi Koike, Satoru Kumaki, Nobuyuki Tanaka, Toshikazu Takeshita

**Affiliations:** 1 Department of Microbiology and Immunology, Shinshu University School of Medicine, 3-1-1 Asahi, Matsumoto, Nagano, 390-8621, Japan; 2 Department of Medical Genetics, Shinshu University School of Medicine, 3-1-1 Asahi, Matsumoto, Nagano, 390-8621, Japan; 3 Department of Obstetrics and Gynecology, Shinshu University School of Medicine, 3-1-1 Asahi, Matsumoto, Nagano, 390-8621, Japan; 4 Department of Pediatrics, Shinshu University School of Medicine, 3-1-1 Asahi, Matsumoto, Nagano, 390-8621, Japan; 5 Department of Pediatrics, Tohoku University Graduate School of Medicine, 2-1 Seiryo-machi, Aoba-ku, Sendai, 980-8575, Japan; 6 Division of Immunology, Miyagi Cancer Center Research Institute, 47-1 Nodayama, Medeshima-Shiode, Natori, Miyagi 981-1293, Japan

## Abstract

Therapeutic retroviral vector integration near the oncogene *LMO2 *is thought to be a cause of leukemia in X-SCID gene therapy trials. However, no published studies have evaluated the frequency of vector integrations near exon 1 of the *LMO2 *locus. We identified a high incidence region (HIR) of vector integration using PCR techniques in the upstream region close to the *LMO2 *transcription start site in the TPA-Mat T cell line. The integration frequency of the HIR was one per 4.46 × 10^4 ^cells. This HIR was also found in Jurkat T cells but was absent from HeLa cells. Furthermore, using human cord blood-derived CD34^+ ^cells we identified a HIR in a similar region as the TPA-Mat T cell line. One of the X-linked severe combined immunodeficiency (X-SCID) patients that developed leukemia after gene therapy had a vector integration site in this HIR. Therefore, the descriptions of the location and the integration frequency of the HIR presented here may help us to better understand vector-induced leukemogenesis.

## Findings

The *IL2RG *gene encodes the interleukin-2 receptor γ chain (IL-2Rγ) [1], and mutations in this gene cause X-linked severe combined immunodeficiency (X-SCID) [2]. Gene therapy trials for X-SCID have achieved remarkably successful outcomes [3-5] but have also been associated with leukemogenesis in some patients. Analyses of leukemic cell clones from these patients revealed that the murine leukemia virus (MLV) vector had integrated proximal to the promoter of an oncogene involved in T-cell acute lymphoblastic leukemia, *LIM-only protein 2 *(*LMO2*), resulting in aberrant expression. These findings suggest that retroviral vector integration near the *LMO2 *promoter is the most likely cause of leukemogenesis in these cases [6-8]. Several oncogenes, including *LMO2*, have very recently been reported to be target genes for vector integration in two patients that developed leukemia following retroviral-mediated gene therapy [9,10]. Accordingly, a determination of the frequency of vector integration near the transcription start site (TSS) of *LMO2 *would be important for understanding the mechanism of the *LMO2 *insertional mutagenesis observed in the leukemic cell clones. The frequency of vector integrations near the TSS of the *LMO2 *locus has not been previously described. In the present study, we have detected a region where vectors integrated with high frequency near the TSS of the *LMO2 *locus in two T cell lines and human cord blood-derived CD34^+ ^cells, and we have subsequently determined the frequency of this vector integration in TPA-Mat and CD34^+ ^cells.

We previously identified 340 integration sites and 15 integration hotspots (defined as ≥ 3 integration sites within a 100-kb region) for MLV vector integration in infected human T cell line clones [11]. A hotspot in intron 2 of the *TRAF2- and NCK-interacting kinase *(*TNIK*) gene had three integration sites within 3.5-kb, indicating that this hotspot is an appropriate locus for estimating the integration frequency. We selected clone 705-9, which has an integrated vector in the hotspot region of the *TNIK *locus [11]. We investigated the sensitivity of the PCR techniques utilized in this study. One copy of the junction sequence between the virus gene and the *TNIK *gene was amplified from DNA harvested from 705-9 cells, in the presence of 1 μg (1.5 × 10^5 ^cells) of genomic DNA from parental TPA-Mat-ecoR cells. A nested PCR using a 3' LTR-specific primer and a *TNIK*-specific primer showed that one copy of the integrated vector was detectable as a 1.5-kb PCR product (data not shown), demonstrating the sensitivity of this assay.

**Figure 1:**
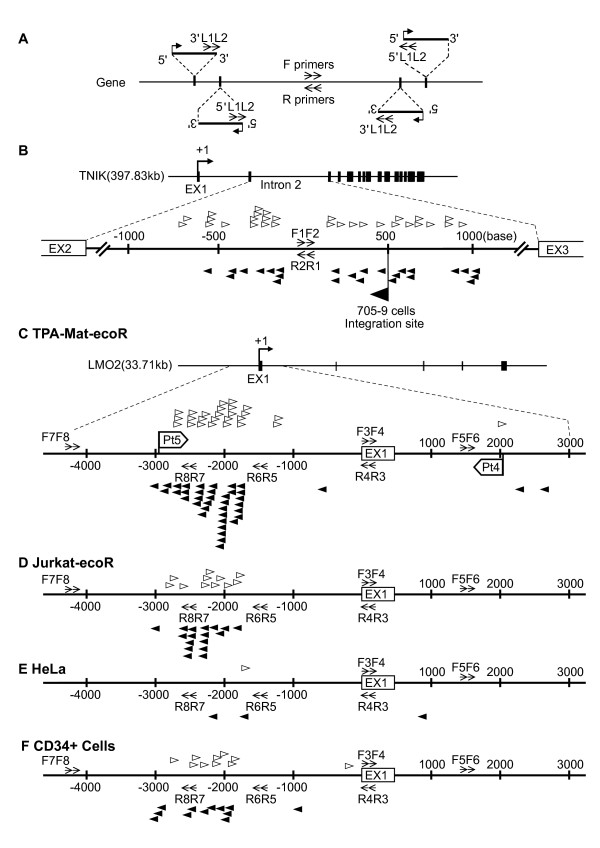
**MLV vector integrations into the *TNIK *and *LMO2 *gene loci**. (A) Schematic representation of MLV vector integration into a gene locus. MLV vector integrations were detected using nested PCR with a combination of 3' or 5' LTR-specific primers (3' L1L2 or 5' L1L2) and gene-specific primers (F or R). (B) MLV integration sites in the integration hotspot of the *TNIK *gene locus. Upper: Diagrammatic representation of the *TNIK *gene locus. Exons and the transcription start site are shown as Ex and +1, respectively. Lower: MLV vectors integrated into an approximately 2-kb region within the *TNIK *hotspot were detected by PCR with combinations of MLV vector-specific primers (3' L1L2 or 5' L1L2) and *TNIK*-specific primers (F1F2 or R1R2), as described in (A). The numbers indicate the nucleotide distance from the *TNIK*-specific primers (F1F2 or R1R2). The PCR products were sequenced, and the locations of the integration sites were determined by use of the human BLAST program. The integration site within the 705-9 cell hotspot was identified in our previous study [11] (large black arrowhead). (C) MLV integration sites near exon 1 of the *LMO2 *gene in TPA-Mat cells. Upper: Diagrammatic representation of the *LMO2 *gene locus. Lower: MLV vectors integrated into an approximately. ± 3-kb region from the transcription start site of *LMO2 *were detected by PCR with combinations of MLV vector-specific primers (3' L1L2 or 5' L1L2) and *LMO2*-specific primers (F3F4, F5F6, F7F8, R3R4, R5R6 or R7R8), as described in (B). The numbers indicate the nucleotide distance from the transcription start site. Pt4 and Pt5 indicate the therapeutic MLV vector integration sites in patients 4 and 5, respectively, who developed leukemia after the French X-SCID gene therapy trials. (D) MLV integration sites near exon 1 of the *LMO2 *gene in Jurkat-ecoR cells. (E) MLV integration sites near exon 1 of the *LMO2 *gene in HeLa cells. (F) MLV integration sites near exon 1 of the *LMO2 *gene in human CD34^+ ^cells. Black and white arrowheads respectively denote the reverse and forward orientation, relative to transcription, of the integrated MLV vectors.

To estimate the integration frequency in a human T cell line, TPA-Mat-ecoR cells expressing the ecotropic mouse receptor were infected with an ecotropic MLV vector that encoded green fluorescent protein (GFP); the infection efficiency (29 - 46%, based on GFP fluorescence) was similar to that measured in patients in the gene therapy trials [12]. At 48 hours post-infection, genomic DNA was isolated from the cells. This acute infection system is suitable for analyzing the distribution of initial vector integration. The combinations of LTR- and *TNIK*- or *LMO2*-specific primers (Additional file 1) used for the PCR reactions are shown in Figure 1A. All resulting PCR products from 186 PCR amplifications (1 μg DNA was used for each PCR amplification, and 186 μg of sample DNA correspond to approximately 2.8 × 10^7 ^cells) carried out using LTR- and *TNIK*-specific primers were cloned and sequenced, and 55 integration sites were mapped within the human genome (Figure 1B and Additional files 2 and 3). For 1613 PCR amplifications (1613 μg of sample DNA corresponds to approximately 2.4 × 10^8 ^cells) using LTR- and *LMO2*-specific primers, 65 integration sites were unevenly distributed in an approximately ± 3-kb region surrounding exon 1 of *LMO2 *in the human genome (Figure 1C and Additional files 2 and 3). We found a high incidence region (HIR) of vector integration in the region upstream of -1740, near the *LMO2 *promoter (Figure 1C). A HIR was also observed around the 705-9 cell integration site of the *TNIK *locus. To confirm the HIR location in the *LMO2 *locus, we performed additional PCR assays in the region upstream of -3000. Since no integration into the region upstream of -3001 was detected with the indicated primers (Figure 1C and Additional files 2 and 3), we suggest that the HIR in the TPA-Mat-ecoR cells ranges from -1740 (the downstream edge) to -3001 (the upstream edge). We observed multiple-hit integrations that included two or three vector integrations at the same nucleotide position within this HIR of *LMO2*, as described below. In contrast, no integration was detected downstream (1 ~1500) of exon 1 (0/270) and only a few integrations were found from 1500 to 3000 (3/270). Subsequent analysis using the same primer sets in a second T cell line, Jurkat-ecoR (infection efficiency: 28-36%, based on GFP fluorescence) identified a HIR (-1801 to -2968) in a similar region as the TPA-Mat-ecoR cells. No other integration sites near the *LMO2 *promoter were detected in Jurkat-ecoR cells (Figure 1D and Additional files 2 and 3).

**Figure 2:**
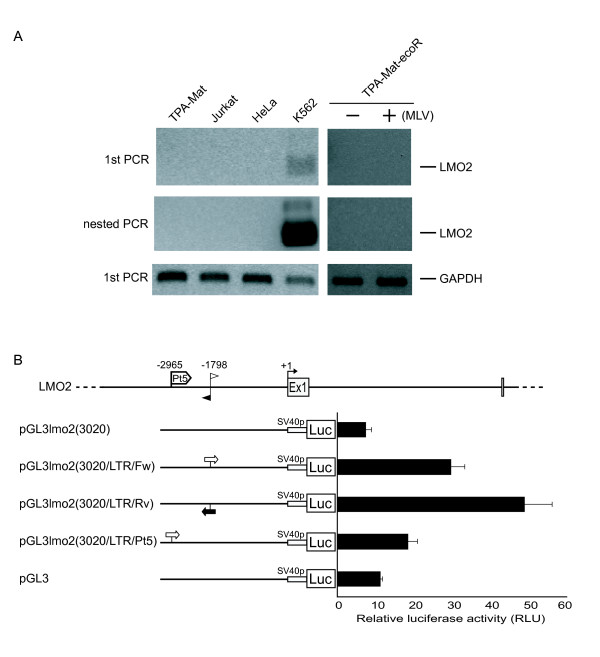
**Endogenous or induced expression of the *LMO2 *gene**. (A) mRNA expression of the *LMO2 *gene in TPA-Mat, Jurkat, HeLa, K562 and TPA-Mat-ecoR cells. Total RNA isolated from the indicated cells was subjected to RT-PCR using the primers for LMO2 or GAPDH as a control. Aliquots of the LMO2 PCR products were subsequently subjected to the nested PCR for LMO2. The PCR products were visualized with ethidium bromide staining. Upper, middle and lower panels indicate the PCR products derived from LMO2, LMO2 and GAPDH mRNA, respectively. TPA-Mat-ecoR cells were infected with (+) or without (-) the MLV vector. (B) Induction of reporter gene activity by the insertion of MLV LTR into the HIR. Luciferase expression constructs with the MLV LTR inserted into the HIR of the *LMO2 *promoter region were assayed in TPA-Mat-ecoR cells. -2965 and -1798 indicate an integration site reported in the leukemia patient and a site where we found forward or reverse orientation integrated vector, respectively. Black and white arrows respectively denote the reverse and forward orientation, relative to transcription, of the integrated MLV LTRs.

In a previous report, we found fewer hotspots in HeLa cells than in TPA-Mat cells [11,13]. In addition, almost none of the genes present in hematopoietic stem cell hotspots were found in HeLa cell hotspots [14], suggesting that hotspots in these different cell types are distinct. We examined vector integration near the TSS of the *LMO2 *locus in HeLa cells to address this point. Only a few integrations were observed upstream (-1 ~-3000; 3/540) and downstream (1 ~3000; 1/540) of exon 1 (Figure 1E, Additional files 2 and 3) despite the high infection efficiency of HeLa cells (45 ~58% based on GFP fluorescence). Real-time PCR showed that the vector integration copy number in TPA-Mat-ecoR cells (infection efficiency; 42%, based on GFP fluorescence) was estimated at 2.0 per diploid genome when normalized to interferon γ DNA, or 2.3 per diploid genome according to the 42% GFP fluorescence in the standard curve based on the real-time PCR analyses (Additional file 4). The vector integration copy number in HeLa cells was estimated at 1.4 per diploid genome according to the 45% GFP fluorescence in the standard curve based on the real-time PCR analyses (Additional file 4). The three upstream integration sites (-1740, -1875 and -2068) in HeLa cells were also found in TPA-Mat-ecoR cells with the -1875 integration site also present in Jurkat-ecoR cells (Additional file 3). In the -1740 and -1875 integration sites of the TPA-Mat-ecoR cells, we observed two and three integrated vectors, respectively. All multiple-hit integrations listed in Additional file 3 were derived from independent infection experiments. A previous study demonstrated that the integration sites of MLV vectors showed a weak favoring of active transcription units [15]. To examine whether endogenous *LMO2 *mRNA-levels correlated with the frequency of vector integration, we analyzed transcription of the *LMO2 *gene in TPA-Mat, Jurkat, HeLa and the LMO2 expressing K562 cells [16]. Reverse transcriptase (RT)-PCR showed that endogenous transcription of the *LMO2 *gene was only detected in K562 cells (Figure 2A). Thus, the frequency of vector integration in TPA-Mat, Jurkat and HeLa cells is not influenced by the endogenous *LMO2 *mRNA-level.

Subsequently, we examined whether vector integration would affect LMO2 expression in TPA-Mat-ecoR cells. Endogenous *LMO2 *mRNA was not detected after vector infection (100%, based on GFP fluorescence) in TPA-Mat-ecoR cells (Figure 2A). We then prepared a series of luciferase reporter gene constructs containing the region between -3020 and +147 of the *LMO2 *promoter region. The construct pGL3lmo2 (3020) containing the region (-3020 ~+147) was virtually silent compared with the pGL3-basic construct containing a SV40 promoter only (Figure 2B). The insertion of the MLV LTR into a site (-1798) within the HIR, where forward or reverse orientation of the inserted vector was observed (Additional file 3), resulted in significant increases in reporter gene activity. A similar result was obtained by the insertion into another site (-2965), which is an integration site reported in the leukemia patient. Consequently, these results suggest that vector integration at -1798 within the HIR may increase transcriptional activity of the *LMO2 *gene, similar to the report for vector integration at -2965 in the leukemia patient [12].

We compared the integration pattern in the TPA-Mat-ecoR cells with the integration sites identified in patients (Pt) 4 and 5 who developed leukemia during the gene therapy trials for treatment of X-SCID [12,17]. Vector integration into the position detected in Pt4 (Figure 1C) was rare in TPA-Mat-ecoR cells; differences in the integration frequencies between the upstream (-1 ~-3000; 60/533) and downstream (1 ~3000; 3/540) regions of exon 1 (Figure 1C) were observed. In contrast, the integration site (-2965) in Pt5 was located in the HIR (-1740 ~-3001) (Figure 1C). Since CD34^+ ^hematopoietic stem cells have been infected with the MLV vector in the clinical trials, we investigated whether the HIR is found in human CD34^+ ^hematopoietic stem cells. Using the same primer sets in umbilical cord blood CD34^+ ^cells (infection efficiency: 14.7%, based on GFP fluorescence), we have identified an HIR (-1882 to -2971) (18/270) in a similar region as the TPA-Mat-ecoR and Jurkat-ecoR cells (Figure 1F, Additional files 2 and 3). Only a few integrations were found from 1 to -1500 (2/270) (Figure 1F, Additional files 2 and 3). Thus, analyzing the location of the HIR in hematopoietic stem cells in these patients will provide insights into leukemogenic integration sites and may have an impact on future gene therapy trials. The HIR is also a suitable region for analyzing the molecular mechanism of vector integration with target-site preferences [14,18]. On the other hand, results showing retroviral integration sites 35 kb upstream [10] and 10.6 kb downstream [9] of the TSS were reported in the patients, and sites 36.3 kb, 69.2 kb, 68.0 kb, 68.3 kb and 0.9 kb upstream of the *LMO2 *TSS were detected in a murine leukemia model [19]. This indicates that integrations in the sites or regions which are far from the TSS are closely associated in *LMO2*-related leukemogenesis. Analysis of the differences and similarities between the HIR near the TSS and the regions far from the TSS will therefore facilitate the elucidation of *LMO2*-related leukemogenesis in the future and may identify additional HIRs that may exist far from the TSS.

**Figure 3:**
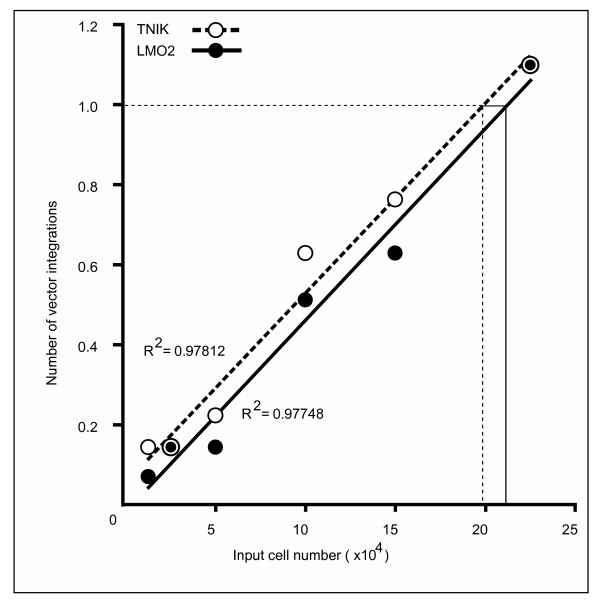
**Vector integration frequencies in the high incidence regions of the *LMO2 *and *TNIK *gene loci**. MLV vectors integrated in the HIR of the *TNIK *(open circles) and *LMO2 *(closed circles) gene loci were detected by PCR with combinations of MLV vector-specific primers (3' L1L2) and gene-specific primers (F1F2 or R5R6) using extracted DNA samples from MLV-infected cells as template. Our results indicate that 42% of these MLV-infected cells expressed GFP. The number of vector integrations represents the number of detected integrations per 15 PCR amplifications, as calculated by Poisson distribution analyses. Each data set gave straight lines fitted by a linear approximation with a correlation coefficient (*TNIK*: R^2 ^= 0.978; *LMO2*: R^2 ^= 0.977). The calculated frequencies, according to each line, were one per 1.992 × 10^5 ^cells (*TNIK*) and 2.125 × 10^5 ^cells (*LMO2*). The frequencies of vector integration into the HIRs of the *TNIK *and *LMO2 *genes, which were calculated using data based on Poisson distribution analyses, were one per 4.18 × 10^4 ^cells (1.992 × 10^5 ^cells (based on Poisson distribution analyses) × 0.42 (% of GFP positive cells)/2 (3' LTR primer direction/3' and 5' LTR primer directions)) and one per 4.46 × 10^4 ^cells(2.125 × 10^5 ^cells (based on Poisson distribution analyses) × 0.42 (% of GFP positive cells)/2 (3' LTR primer direction/3' and 5' LTR primer directions)), respectively.

We have attempted to estimate the number of cells that carried an integrated vector in the HIR near exon 1 of the *LMO2 *locus in the leukemia patients who participated in the gene therapy trials for treatment of X-SCID [12]. The integration frequencies calculated from the HIR data on the *TNIK *and *LMO2 *loci were one per 4.18 × 10^4 ^cells and one per 4.46 × 10^4 ^cells (or one per 1.992 × 10^5 ^integrations and one per 2.125 × 10^5 ^integrations), respectively (Figure 3, Additional files 4 and 5). This estimate was calculated using the integration frequency data for the HIR that was obtained in the TPA-Mat-ecoR cells as calculated in Figure 3. 133 × 10^6 ^transduced cells were infused into Pt5 [12] in the gene therapy trial. Given that at least 1% of the transduced cells could give rise to T cells [12], Pt5 would have received 30 HIR-targeted cells, which suggests that the frequency of vector integration in the HIR found in TPA-Mat-ecoR cells may have contributed to the observed leukemogenesis in Pt5 in the X-SCID gene therapy trial. Furthermore, we have attempted to estimate the integration frequency of CD34^+ ^cells. The integration frequency of CD34^+ ^cells calculated from the HIR data based on the vector integration copy number of CD34^+ ^and TPA-Mat cells was estimated to be one per 9.00 × 10^4 ^integrations or one per 1.89 × 10^4 ^cells (Additional file 5). Our estimation for the integration frequency of the HIR suggested that a patient has a substantial chance that the transfused cells would have the vector integration in the HIR near the *LMO2*. However, not every patient develops leukemia and leukemia development takes several years to occur. Additional factors, such as mutations in other T-cell oncogenes or additional insertional mutagenesis, can contribute to leukemogenesis and were observed in the leukemia patients [12] and murine leukemia model [19,20]. Although there is controversy about the contribution of the therapeutic *IL2RG *gene in the leukemogenesis [6,7,19], if the activation of only two genes, *LMO2 *and the *IL2RG*, were enough to induce leukemia, more of the patients would have developed leukemia in light of our estimation for the integration frequency and the reports that there are hotspots in the *LMO2 *locus [14,21]. Thus three or four factors may be needed for leukemogenesis, and the use of retroviral vectors without a tendency to form HIRs near the *LMO2 *locus may improve the safety of gene therapy.

In conclusion, the identification of the HIR near exon 1 of the *LMO2 *locus in the T cell lines and human CD34^+ ^cells may partially explain the mechanism responsible for the *LMO2*-insertional mutagenesis observed in leukemic cell clones. It may help us to better understand vector-induced leukemogenesis.

## Competing interests

The authors declare that they have no competing interests.

## Authors' contributions

KYa, TTs and KYo designed and performed the research and wrote the manuscript. KK performed the research, analyzed the experimental conditions and wrote the manuscript. RO and TS analyzed the experimental conditions including the collection of human cord blood, KS and KK analyzed the experimental conditions including the purification of CD34^+ ^cells. HA, YY, YA, NK, MH, KW and YF performed some of the experiments, generated research tools and participated in discussions. NT and SK provided critical advice. TTa wrote the final manuscript.

## Supplementary Material

Additional file 1 **Sequences of LTR, TNIK, and LMO2 primer sets.** Click here for file

Additional file 2 **MLV integration sites in the TNIK and LMO2 loci.** Click here for file

Additional file 3 **Positions of the integration sites near the LMO2 and TNIK loci.** Click here for file

Additional file 4 **Standard curve of the relationship between the percentage of GFP-positive cells and the vector copy-number per genome.** Click here for file

Additional file 5 **Materials and Methods.** Click here for file
