# Supplementary material for: Identification of a high incidence region for retroviral vector integration near exon 1 of the LMO2 locus
Source: Retrovirology. 2009 Sep 2;6:79. doi: 10.1186/1742-4690-6-79 (PMC2742512; doi:10.1186/1742-4690-6-79)
Supplement: Additional file 4 — Standard curve of the relationship between the percentage of GFP-positive cells and the vector copy-number per genome. [file 1742-4690-6-79-S4.pdf]

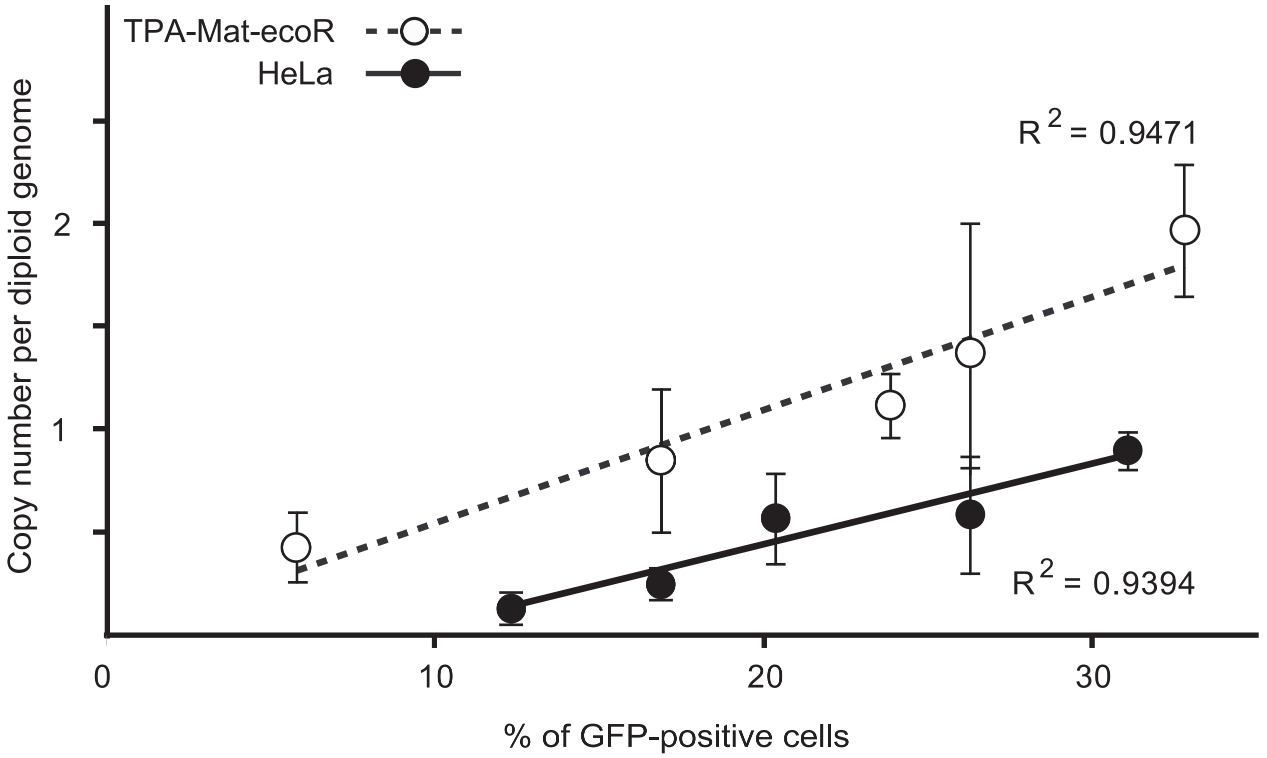

Addtional File 4. Standard curve of the relationship between the percentage of GFP-positive cells and the vector copy-number per genome

Supernatants containing the MLV vector were serially diluted and mixed with TPA-Mat-ecoR and HeLa cells. 48hours later, the cells were harvested and assessed according to the GFP fluorescence by flow cytometry. Genomic DNA was purified from the cells and subjected to quantitative PCR analysis with ABI Prism 7000 Real Time PCR system.
