# Supplementary material for: Identification of a high incidence region for retroviral vector integration near exon 1 of the LMO2 locus
Source: Retrovirology. 2009 Sep 2;6:79. doi: 10.1186/1742-4690-6-79 (PMC2742512; doi:10.1186/1742-4690-6-79)
Supplement: Additional file 5 — Materials and Methods. [file 1742-4690-6-79-S5.pdf]

Materials and Methods

Plasmids

An expression vector for pcDNAV5-ecoR was constructed by ligating a cDNA fragment encoding the mouse ecotropic receptor from pM5neo [1] into the *XbaI* and *XhoI* sites of pcDNA3.1/V5-His-A (Invitrogen, Carlsbad, CA, USA), which contains a neomycin-resistance gene. The pMXs-GFP vector, a Moloney murine leukemia virus-derived MLV vector that contains GFP, and a packaging plasmid, gagpol-IRES-brs, were also used [2, 3]. The pcDNA VSV-G plasmid, encoding a vesicular stomatitis virus glycoprotein, was kindly provided by Dr. T. Kafri [4].

Cell lines and transfection

TPA-Mat cells, derived from a human T-cell leukemia virus type 1-infected T-cell line [5], were maintained in RPMI 1640 supplemented with 10% fetal calf serum (FCS) and 200 nM phorbol-12,13-dibutyrate (PDBu; Sigma, St. Louis, MO, USA) at 37°C with 5% CO_2. Jurkat cells were maintained in the same cell culture medium without PDBu. TPA-Mat and Jurkat cell clones stably expressing the ecotropic mouse receptor were established by neomycin-selection after gene transfection as described below. TPA-Mat or Jurkat cells (5×10^6) suspended in

0.5 ml of Opti-MEM (Invitrogen, Carlsbad, CA, USA) were transfected with 10 μg of pcDNAV5-ecoR by electroporation at 250 V and 950 μF with a Gene Pulser (Bio-Rad, Hercules, CA, USA). At 6 hours after transfection, the cells were seeded in 96-well plates and selected in medium containing G418 (0.8 mg/ml) for 2 weeks. Single-cell G418-resistant clones were screened for sensitivity to MLV infection using the MLV vector containing GFP. The clone showing the highest sensitivity to MLV infection was selected and designated TPA-Mat-ecoR or Jurkat-ecoR. HeLa, 293T, and Plat-E cells were maintained in DMEM supplemented with 10% FCS at 37°C with 5% CO_2.

Preparation of MLV vector and infection

To produce recombinant ecotropic MLV viruses containing GFP, Plat-E cells (1×10^6) were seeded in 10-cm dishes, incubated for 24 hours and transfected with 10 μg of pMXs-GFP by using a calcium-phosphate precipitation method. After 12 hours of incubation, the culture media was exchanged for fresh media. The supernatants were harvested 48 hours after transfection and filtered with 0.45-μm pore-size membrane filters (Millipore, Billerica, MA, USA). Next, TPA-Mat-ecoR or Jurkat-ecoR cells (1×10^6) were mixed with 0.5 ml of the

supernatants and diluted with an equal volume of RPMI medium containing 4 μg/ml of polybrene with or without 200 nM PDBu. The cells were then transferred to 12-well plates and centrifuged at 2,200 rpm for 1 hour at 30°C. After 12 hours of incubation, the culture media was exchanged for fresh media. 48 hours after infection, the cells were harvested, and fixed with 1% paraformaldehyde in PBS. The infection efficiencies of TPA-Mat-ecoR and Jurkat-ecoR cells were assessed according to the GFP fluorescence by flow cytometry using a FACSCalibur (Becton Dickinson, San Jose, CA, USA). In order to produce recombinant MLV viruses that can infect HeLa or human $CD34^+$ cells, 293T cells (1×10^6) were transfected with 10 μg each of gagpol-IRES-brs, pMXs-GFP and 5 μg of pcDNA VSV-G by a calcium-phosphate precipitation method.

Transduction of human $CD34^+$ cells

As reported previously [6], $CD34^+$ cells, 92.2% of which was $CD34^+$-positive by flow cytometry analysis (FACSCalibur; Becton Dickinson, San Jose, CA, USA), were purified from umbilical cord blood Ficoll fractions by magnetic sorting (Direct CD34 Progenitor Cell Isolation Kit; Miltenyi, Auburn, CA,USA) and pre-stimulated for 24 hours in X-VIVO 10 medium (Lonza Walkersville. Inc,

Walkersville, MD, USA) supplemented with 4% FCS in the presence of 100ng/ml human thrombopoietin (TPO), 300ng/ml Flt3/Flk2 ligand, 60ng/ml IL-3, and 300ng/ml human stem-cell factor (R&D systems, Minneapolis, MIN, USA). The cells were then transduced with viral supernatant (MOI=30) by spinoculation (twice, as described above) in the presence of polybrene (4μg/ml) and incubated for 8 days. The infection efficiency was 14.7% based on GFP fluorescence. This study was approved by the Ethics Committee of the Shinshu University School of Medicine, and written informed consent was obtained from each healthy donor.

Cloning of MLV integration sites

To identify MLV vector integration sites near exon 1 of the LMO2 gene or integration hotspots in the TRAF2- and NCK-interacting kinase (TNIK) gene, genomic DNA was isolated from infected TPA-Mat-ecoR cells, Jurkat-ecoR cells, HeLa cells or $CD34^+$ cells using a proteinase K extraction method. The polymerase chain reaction (PCR) conditions were denaturation at 94°C for 2 minutes, followed by 9 cycles of denaturation at 94°C for 20 seconds, annealing at 65°C for 20 seconds graduating by -0.5°C per cycle and extension at 72°C for

45 seconds, and 30 cycles of 94°C for 20 seconds, 65°C for 20 seconds and 72°C for 45 seconds. Next, 1/100 of the volume of the first-round PCR product was subjected to a second round of PCR under the same PCR conditions with nested primers. The amplified PCR products were separated in 1.5% agarose gels containing ethidium bromide, cloned into the p3T vector (MoBi Tec, Goettingen, Germany), transformed into DH5α and sequenced in an ABI PRISM 3100 Genteci Analyzer (Applied Biosystems, Foster City, CA, USA).

Mapping of MLV integration sites

MLV vector integration sites were mapped to the human genome as described previously [2]. The raw sequences were trimmed to remove all vector elements, and the remaining portion of the sequences was reviewed. The junction sequences next to the 3' or 5' long terminal repeat (LTR) end sequences were compared with the human genome using the BLAST program (http://blast.ncbi.nlm.nih.gov/Blast.cgi). Authentic integration sites used in the analysis were defined as: (i) containing a sequence contiguous from the nested primer for the LTR (e.g., the end of the 3' or 5' LTR) to the nested primer sequence in the genome (e.g., LMO2 or TNIK locus); (ii) matching a genomic

location starting immediately (within three bases) after the end of the 3' or 5' LTR; and (iii) showing 96% or higher identity with the human genome.

The BLAST program and Ensemble genome browser (http://www.ensembl.org/index.html) were used to determine the locations of matches with respect to exons and introns.

RT-PCR

We analyzed the mRNA-levels of the LMO2 gene in the uninfected TPA-Mat, Jurkat, HeLa, K562 and TPA-Mat-ecoR cells or in the infected TPA-Mat-ecoR cells by a reverse transcriptase RT-PCR as described previously [2]. The primers used in RT-PCR were as follows:

LMO2

1st PCR Fw: 5’-ATGTCCTCGGCCATCG-3’

1st PCR Rv: 5’-CATCGAAAGGAAGAGCCTGG-3’,

nested PCR Fw: 5’-GACCGTCTTGCCCAAAAAGC-3’

nested PCR Rv: 5’-TCCGGCCCAGTTTGTAGTAG-3’

GAPDH

Fw: 5’- ACCACAGTCCATGCCATCAC-3’

Rv: 5'- TCCACCACCCTGTTGCTGTA-3'

The LMO2 PCR programs were 94°C for 5 min, followed by 35 cycles of 94°C for 30 s, 62°C for 1 min, and 72°C for 2 min. Nested PCR used 1/100 volume of the first round PCR product and was performed under the same PCR cycle conditions with nested primers. GAPDH was used as an internal control under the following program, 94°C for 5 min, followed by 20 cycles of 94°C for 30 s, 58°C for 1 min, and 72°C for 2 min. The PCR products were separated on 2% agarose gels, visualized by ethidium bromide staining, and also subcloned into a p3T cloning vector (Bio-Rad Laboratories, USA) and subjected to nucleotide sequence analysis to verify their identity.

Luciferase promoter assay

The promoter region of LMO2, corresponding to -3020 to +147 bp (relative to the LMO2 transcription start site, +1), was amplified by PCR using TPA-Mat genomic DNA as template, and cloned into *MluI* and *XhoI* sites of pGL3-basic plasmid containing a SV40 promoter (Promega Madison, WI USA), and designated pGL3lmo2(3020) as previously described [7]. To assess the influence of MLV vector insertion in the high incidence region (HIR) of the *LMO2* gene, the LTR of

MLV was inserted at positions –2965 or -1798 in these constructs. The resulting plasmids were designated pGL3lmo2(3020/LTR/Pt5), which contains the LTR at position -2965, and pGL3lmo2(3020/LTR/Fw) and pGL3lmo2(3020/LTR/Rv), which contains the LTR at position -1798, in either a forward or reverse orientation, respectively. Genomic location of the *LMO2* gene is based on the UCSC genome browser (as of July 2003). The reporter assays were performed as described in the cell line section. Briefly, TPA-Mat cells ($5x10^6$) were co-transfected with 10 μg of the LTR-containing reporter plasmid, and 0.2 μg of a PRL-null plasmid (Promega, Madison, WI, USA), which served as an internal control for electroporation. 48hr post transfection, cell lysates were prepared according to the manufacturer's protocol for the Dual-Luciferase Reporter Assay System (Promega, Madison, WI, USA). Relative luciferase activity (RLU) are the ratios between firefly-luciferase (expressed from the LTR- containing plasmids) and Renilla-luciferase (PRL-null plasmid).

Real-time PCR

To estimate copy number of the integrated MLV vector per genome, we performed quantitative PCR analysis for TPA-Mat-ecoR, HeLa and $CD34^+$ cells.

The genomic DNA was separated from the episome-type vector of the cytoplasm by 1% low-melting-point agarose gel electrophoresis, and using the primers of MLV and human interferon γ as an endogenous reference (Provirus Copy Number Detection Primer Set, human; Takara Bio Inc, Otsu, Japan) quantitative PCR analysis was performed with an ABI Prism 7000 Real Time PCR system (Applied Biosystems, Foster City, CA, USA). The PCR program was 95°C for 30 seconds, followed by 40 cycles of 95°C for 5 seconds, 55°C for 15 seconds, and 72°C for 31 seconds. To estimate difference in the amplification efficiency of the MLV vector to interferon γ, we prepared serial dilutions of the standard plasmid (containing one copy of MLV and interferon γ DNA partial sequence) of the Provirus Copy Number Detection Primer Set, and then MLV DNA values were normalized against endogenous interferon γ DNA values that were corrected according to the difference in amplification between the MLV vector and interferon γ as described above.

Estimation of the integration frequency

We performed the Poisson distribution analysis and linear approximation with a correlation coefficient using Microsoft Excel 2003. MLV vectors integrated in the

HIR of the *TNIK* and *LMO2* gene loci were detected by PCR with combinations of MLV vector-specific primers (3' L1L2) and gene-specific primers (F1F2 or R5R6) using extracted DNA samples from MLV-infected cells as template. Our results indicate that 42% of these MLV-infected cells expressed GFP. The number of vector integrations represents the number of detected integrations per 15 PCR amplifications, as calculated by Poisson distribution analyses. The principle of dilution analysis used in this study is that cells which have the integrated vector in the HIR of the *LMO2* are randomly distributed in the diluted cell suspension, and each vector integrated cell has an equal opportunity to be redistributed into the diluted cell suspension (according to the 15 samples for PCR amplification). A diluted cell suspension (according to the 15 samples for PCR amplification) may receive any given number (i) of the vector integrated cells with the probability that the diluted cell suspension receives (i) vector integrated cells given by the Poisson equation:

$Pi = e^{-m}(m^i/i!)$.

Here, m is defined as the expected value of the vector integrated cells that are redistributed to a cell suspension (according to 15 samples for PCR amplification). The probability or proportion of samples (aliquots of the cell

suspension) that do not receive a vector integrated cell is represented by the zero term (i = 0) of Poisson distribution:

$P_0 = e^{-m} = 1/ e^m$.

For example, in this case (input cell number; 2.25×10^5 cells), given that 10 (positive samples)/15 (samples; each sample corresponds to 2.25×10^5 cells) could be observed,

P_0 would be approximately 1.0986 as follows:

$P_0 = 1/ e^m = 5$ (negative samples)/15,

$e^m = 15/5$,

$m = \ln 15 - \ln 5 =$ ca. 1.0986

Each data set gave straight lines fitted by a linear approximation with a correlation coefficient (*TNIK*: $R^2 = 0.978$; *LMO2*: $R^2 = 0.977$). The calculated frequencies, according to each line, were one per 1.992×10^5 cells (*TNIK*) and 2.125×10^5 cells (*LMO2*). The frequencies of vector integration into the HIRs of the *TNIK* and *LMO2* genes, which were calculated using data based on Poisson distribution analyses, were one per 4.18×10^4 cells (1.992×10^5 cells (based on Poisson distribution analyses) $\times$ 0.42 (% of GFP positive cells) / 2 (3' LTR primer direction / 3' and 5' LTR primer directions)) and one per 4.46×10^4 cells ($2.125\times$

10^5 cells (based on Poisson distribution analyses) × 0.42 (% of GFP positive cells) / 2 (3' LTR primer direction / 3' and 5' LTR primer directions)), respectively. On the other hand, the frequencies of vector integration into the HIRs of the *TNIK* and *LMO2* genes against the total integrated vectors were one per 1.992×10^5 integrations (1.992×10^5 cells (based on Poisson distribution analyses) × 2.0 (copy number per diploid genome) / 2 (3' LTR primer direction / 3' and 5' LTR primer directions)) and one per 2.125×10^5 integrations (2.125×10^5 cells (based on Poisson distribution analyses) × 2.0 (copy number per diploid genome) / 2 (3' LTR primer direction / 3' and 5' LTR primer directions)), respectively.

To investigate the integration frequency of the HIR based on the Poisson distribution, we selected the 3'L1L2/R5R6 primer set (3' LTR primer direction) as follows.

(according to the table from Additional file 2)

Primer set	Cell	Integrations	Sample
3'L1L2/R5R6	TPA-Mat	23	135μg
5'L1L2/R5R6	TPA-Mat	32	135μg

1) The null hypothesis that there is no difference between 23 integrations (3'L1L2/R5R6) and 32 integrations (5'L1L2/R5R6) was not rejected according to the χ^2 test ($p > 0.1$).
2) To avoid an overestimation of the integration frequency, we selected 3'L1L2/R5R6 (3' LTR primer direction).

Therefore, the integration frequency derived from one primer set (3'L1L2/R5R6) is half a correct value and was doubled.

Estimation of the integration frequency in $CD34^+$ cells

We tried to estimate the integration frequency in $CD34^+$ cells. We first performed real-time PCR to estimate the vector integration copy number in $CD34^+$ cells. The PCR analyses showed that the number of vector integrations in $CD34^+$ cells (infection efficiency; 14.7% based on GFP fluorescence) was estimated at 0.32 per diploid genome.

We calculated the value of the vector integrations in the HIR (primer set;

3’L1L2/R5R6 and 5’L1L2/R5R6) per total integrated vector in $CD34^+$ or TPA-Mat cells according to the data from Additional file 2.

Primer set	Cell	Integrations	Sample	Copy number
3’L1L2/R5R6, 5’L1L2/R5R6	$CD34^+$	9 + 9 = 18	135 + 135 = 270μg	0.32
3’L1L2/R5R6, 5’L1L2/R5R6	TPA-Mat	23 + 32 = 55	135 + 135 = 270μg	2.3

(270μg genomic DNA corresponds to 4.05×10^7 cells)

$CD34^+$ cells:

0.32 (vector copy number) $\times$ 4.05×10^7 cells = 1.296×10^7 (total integrations)

18 (integrations in the HIR)/(1.296×10^7 total integrations) = 1.39×10^{-6}

TPA-Mat cells:

2.3 (vector copy number) $\times$ 4.05×10^7 cells = 9.315×10^7 (total integrations)

55 (integrations in the HIR)/(9.315×10^7 total integrations) = 5.90×10^{-7}

$(1.39 \times 10^{-6})/(5.90 \times 10^{-7}) = 2.36$

Therefore, the integration frequency of the HIR in $CD34^+$ cells is estimated to be 2.36 times higher than that of TPA-Mat cells on the basis of real-time PCR. Accordingly, the integration frequency of the HIR in $CD34^+$ cells was estimated to be one per 9.00×10^4 integrations (2.125×10^5 integrations/2.36) or one per 1.89×10^4 cells (4.46×10^4 cells/2.36).

Supplementary method references

1. Baker BW, Boettiger D, Spooncer E, Norton JD: **Efficient retroviral-mediated gene transfer into human B lymphoblastoid cells expressing mouse ecotropic viral receptor.** *Nucleic Acids Res* 1992, **20:**5234.
2. Tsukahara T, Agawa H, Matsumoto S, Matsuda M, Ueno S, Yamashita Y, Yamada K, Tanaka N, Kojima K, Takeshita T: **Murine leukemia virus vector integration favors promoter regions and regional hot spots in a human T-cell line.** *Biochem Biophys Res Commun* 2006, **345:**1099-1107.
3. Kitamura T, Koshino Y, Shibata F, Oki T, Nakajima H, Nosaka T, Kumagai H: **Retrovirus-mediated gene transfer and expression cloning: powerful tools in functional genomics.** *Exp Hematol* 2003, **31:**1007-1014.
4. Kafri T, Blomer U, Peterson DA, Gage FH, Verma IM: **Sustained expression of genes delivered directly into liver and muscle by**

lentiviral vectors. *Nat Genet* 1997, **17:**314-317.

5. Takeshita T, Goto Y, Nakamura M, Fujii M, Iwami M, Hinuma Y, Sugamura K: **Phorbol esters can persistently replace interleukin-2 (IL-2) for the growth of a human IL-2-dependent T-cell line.** *J Cell Physiol* 1988, **136:**319-325.
6. Cattoglio C, Facchini G, Sartori D, Antonelli A, Miccio A, Cassani B, Schmidt M, von Kalle C, Howe S, Thrasher AJ, et al: **Hot spots of retroviral integration in human CD34+ hematopoietic cells.** *Blood* 2007, **110:**1770-1778.
7. Hammond SM, Crable SC, Anderson KP: **Negative regulatory elements are present in the human LMO2 oncogene and may contribute to its expression in leukemia.** *Leuk Res* 2005, **29:**89-97.
